# Antiseptic Treatment of Gunshot Wound of the Knee-Joint

**Published:** 1880-08-20

**Authors:** J. A. Holloway

**Affiliations:** La.


### ANTISEPTIC TREATMENT OF GUNSHOT WOUND OF THE KNEE-JOINT.

BY J. A. HOLLOWAY, M.D., OF LA.

I was called to see Albert Davis, colored, on the 5th of January, who had been shot during the night previous in the lower part of the thigh with thirty-nine small buckshots—one shot penetrating the knee-joint—the patient remarking soon after my arrival that "all of the oil had run out of the joint."

As the shot were small, I thought it best not to disturb the one entering the joint, but removed those which had entered the other part of the leg and which had passed almost through the leg, and lay just under the skin on the opposite side. He asked me not to amputate, so I resorted to the following antiseptic treatment.

The wounds were kept dressed with carbolized oil, and over the lint, I used towels wetted with carbolized water. I gave three drops of veratrum viride and three grains of salicylic acid every two hours; kept his bowels gently opened.

On the morning of the 8th, patient had very high fever accompanied with very irritable stomach. In three days fever subsided, and stomach became quiet and nourished well. There was very little inflammation about the wounds, and in one month from the date of wounds he was up and walking about on crutches.

He lost the use of tibialis anticus muscle for three or four months, but recovered from that with no other injury to the joint, and can do now any kind of labor.

